# Reproductive Potential Accelerates Preimaginal Development of Rebel Workers in *Apis mellifera*

**DOI:** 10.3390/ani11113245

**Published:** 2021-11-13

**Authors:** Aneta Strachecka, Krzysztof Olszewski, Karolina Kuszewska, Jerzy Paleolog, Michał Woyciechowski

**Affiliations:** 1 Department of Invertebrate Ecophysiology and Experimental Biology, University of Life Sciences in Lublin, 20-950 Lublin, Poland; jerzy.paleolog@up.lublin.pl; 2 Institute of Biological Basis of Animal Production, Faculty of Animal Sciences and Bioeconomy, University of Life Sciences in Lublin, Akademicka 13, 20-950 Lublin, Poland; krzysztof.olszewski@up.lublin.pl; 3 Institute of Environmental Sciences, Jagiellonian University, 30-387 Krakow, Poland; k.kuszewska@gmail.com (K.K.); michal.woyciechowski@uj.edu.pl (M.W.)

**Keywords:** *Apis mellifera*, rebel workers, reproductive potential, preimaginal development

## Abstract

**Simple Summary:**

All female honeybee larvae may develop into workers or queens, depending on the food they receive. During this period, queen mandibular pheromones (QMP) perform a regulatory function in inhibiting ovarian development in adult workers. These pheromones are transmitted (via trophallaxis) by workers to pass information to larvae on the presence or absence of the queen. Queen-less conditions are conducive to the emergence of rebel workers that are set to reproduce, and do not participate in the rearing of successive bee generations in contrast to the sterile, normal workers. We posited that rebels are not only similar to queens in some anatomical features, but also develop in a shorter time in comparison to normal workers. Therefore, the aim of this study was to compare the duration of preimaginal development in rebel and normal workers. Our results confirmed that the workers who develop in a queen-less colony undergo a shorter preimaginal development than those in a queen-right colony.

**Abstract:**

Rebel workers develop from eggs laid by the previous queen, before it went swarming and left the colony orphaned, until the emergence of a new queen. In contrast to normal workers developing in the queen’s presence, rebels are set to reproduce and avoid rearing of successive bee generations. They have more ovarioles in their ovaries, as well as more developed mandibular glands and underdeveloped hypopharyngeal glands, just like the queen. We posited that rebels are not only similar to queens in some anatomical features, but also develop in a shorter time in comparison to normal workers. Therefore, the aim of this study was to compare preimaginal development duration in rebel and normal workers. The results show that rebels, i.e., workers with a higher reproductive potential, had a significantly shorter preimaginal development period (mean ± SD, 19.24 ± 0.07 days) than normal workers (22.29 ± 0.32 days). Our result confirmed that workers who develop in a queen-less colony undergo a shorter preimaginal development than those in a queen-right colony.

## 1. Introduction

Honeybees (*Apis mellifera*), like other holometabolic insects, have three different ontogenetic phases (larva, pupa, and imago) separated by metamorphic moults. The duration of each of the phases is characteristic of the specified honeybee phenotype, despite the fact that they are produced from the same genomes [1,2]. Female larvae of *A. mellifera* receive different food to become either queens or workers, i.e., those in queen cells are provisioned with royal jelly, while those in worker cells are provided with worker jelly, which includes pollen [3,4,5,6]. However, up to 3 days after hatching, female larvae are totipotent and can develop into queens or workers. Therefore, the 3rd and 4th day of larval development is the crucial period in female honeybee ontogeny [2,7,8]. An important role in this key period is played by mandibular pheromones (QMP), which have a regulatory function in inhibiting ovarian development in adult workers and suppressing their reproduction [9]. These pheromones are transmitted via trophallaxis by workers, and pass information to larvae on the presence or absence of a queen. This information can then change the developmental strategy of these larvae [10]. Usually, the queens take less time to develop (15–16 days) than the workers (21 days) [11].

Swarming is a natural process in the life of a honeybee colony, enabling colonies to multiply. During swarming, the old queen leaves the nest accompanied by a group of workers to establish a new nest/colony, while the remaining workers remain in the old nest to care for the eggs, larvae and pupae of younger workers and new, developing sister queens. New virgin queens begin to emerge approximately a week after the prime swarm has issued, and the first few virgin queens very often leave the nest when the after-swarm has issued [12]. The time between the issuing of the prime swarm and the establishment of the new egg-laying queen is approximately 3–4 weeks [13]. During this time, in queen-less conditions, rebel workers emerge that are phenotypically more queen-like, in contrast to the sterile, normal workers, are set to reproduce and do not participate in the rearing of successive bee generations [14]. They have more ovarioles in their ovaries as well as more developed mandibular glands and underdeveloped hypopharyngeal glands. Moreover, their ovaries are activated regardless of whether they live in queen-less or queen-right colonies [14,15,16,17,18]. Moreover, rebels typically have prolonged life [16], different foraging preferences [18], tendency to drift to other colonies [17], increased sucrose sensitivity [19], increased energy reserves and protein concentrations in the fat body [20], and increased learning ability [21]. We posited that rebels are not only similar to queens in some anatomical, morphological and behavioral features, but also develop in a shorter time in comparison to normal workers, i.e., they might undergo shorter ontogeny. Therefore, the aim of our research study was to determine the duration of preimaginal development of *A. mellifera* rebels. The determination of this in rebels and comparing it to preimaginal development duration in normal workers is the first step in identifying the duration of individual developmental stages, which in turn are crucial for understanding the phenomenon of phenotypic plasticity [2].

## 2. Materials and Methods

This study was performed in June and July 2018 at the apiary of the University of Life Sciences in Lublin, Poland (51°13′26.5404″ N, 22°38′4.7364″ E).

### 2.1. Preparing of Section Combs

Eggs of similar age (not older than 8 h) were obtained using previously unused section combs with dimensions of 115 mm × 95 mm (1.09 dm^2^). On one side of each section comb, a queen excluder was nailed to the frame bar, making the cells on this side too shallow for brood rearing, but allowing the workers to effectively heat the brood on the other side. On the other side of this comb, a frame made of 10 mm × 10 mm thick bars was placed with a queen excluder nailed to it. This allowed the cells to have the correct depth, the bees had free access to them, and the queen caged within the section comb was unable to leave it. Eight such section combs were prepared; four (C1) were used to obtain normal workers and four (C2) to obtain rebel workers.

### 2.2. Experimental Design

In each of the 4 unrelated *Apis m. carnica* source colonies, which populated a two-box hive (Dadant Blatt; 20 frames; 435 mm × 150 mm), a queen was allowed to lay eggs within the section comb (C1) for 8 h. Each box had its entrance. The section combs were placed in the spaces cut in the center of the nest combs. After that time, section comb C1 was transferred from the top box to the bottom box, and the next section comb—C2—was placed in the next nest comb so that the queen could lay eggs again for the next 8 h. The date and time were recorded on section combs C1 and C2. Afterwards, each of the colonies was divided in two equal parts, each in a separate box according to Woyciechowski and Kuszewska [14]. The first part (bottom box), containing the queen, workers, brood, and C1, were used to rear normal (non-rebel) workers, whereas the other part (top box), without a queen but with workers, brood, and C2, served for rearing rebel workers. In order for the foragers not to confuse the boxes, the entrances were closed for one day after separating them. The development of new larvae was prevented in the queenright colony, as described by Woyciechowski and Kuszewska [14]. After 9–10 days, when the larval cells in C1 and C2 were sealed, the two boxes were put together again, respectively, so as to restore each of the 4 source colonies. 

After 18 days from the moment the eggs were laid, four brood section combs were transferred into each of the two glazed observation crates, respectively. These crates were placed in an environmentally controlled incubator (temperature 34.5 °C, relative humidity 60%). In order to record the emergence of bees, the brood section combs were filmed with a Sony DCR-VX2100E camera (Sony Corporation, Minato, Tokyo, Japan). The automatic option of periodic recording was used and the recording time of 2 s alternated with a break time of 5 min. The use of the internal clock of the camera allowed for precise determination of the moment of worker emergence, significantly saving recording time and footage analysis effort. Based on the film, we determined the dates and times at which the first workers emerged, as well as the dates and times at which 50% of the workers emerged (median decomposition). To estimate the exit time point of 50% of the workers, at the initial moment of the recording—when no bees had yet emerged—the sealed brood cells were counted on the monitor screen. Additionally, the arrangement of these cells was marked on a rigid foil glued to the monitor screen. Then, based on this template and counting the cells from which the workers had not yet emerged, the exit time of 50% of the workers was determined. The numbers of sealed brood cells were 315, 286, 273, and 304 in the four normal workers section combs and 307, 316, 251, and 291 in the rebel workers section combs, respectively. After subtracting the date and time of the first workers’ emergence from the date and time of the queen’s caging on the comb sections, the total durations of the preimaginal stages of the individual development of rebel and normal workers were obtained. The time period between the caging of the queen in a given comb section and the emergence of 50% of the workers was also calculated. In order to confirm whether the emerging bees were rebels or normal, Woyciechowski and Kuszewska’s [14] method was used to determine the number of ovarioles (ovarian tubules), as well as the sizes of hypopharyngeal and mandibular glands. Hypopharyngeal and mandibular glands were dissected from the head in physiological salt. The size of the hypopharyngeal gland was calculated from the average diameters of 20 acini (square root of longest × shortest diameters (Figure S1) of 10 acini from right gland and 10 from left gland; the hypopharyngeal gland consists of a great number of lobes, called acini, and their diameter is used routinely as an index of the gland size [14]). Mandibular gland size was calculated from the average of left and right glands (square root of longest × shortest diameters). 

### 2.3. Statistical Analysis

The results were analyzed statistically using Statistica software, version 13.3 (2017) for Windows, StatSoft Inc., Tulsa, Oklahoma, OK, USA. The distribution of the data was analyzed with the Shapiro–Wilk test. The influence of the colony in which brood developed on, namely, the number of ovarioles and sizes of hypopharyngeal and mandibular glands, was assessed separately for rebel and normal workers and together for both groups by the Kruskal–Wallis test, with the exception of sizes of mandibular glands in the rebel workers group where ANOVA was used.

Statistical significance of differences between the duration of the preimaginal stages, as well as the time of emergence of 50% of the workers between rebel and non-rebel workers and their anatomical features, were assessed with the Mann–Whitney test for two in-dependent variables.

## 3. Results

**Figure 1:**
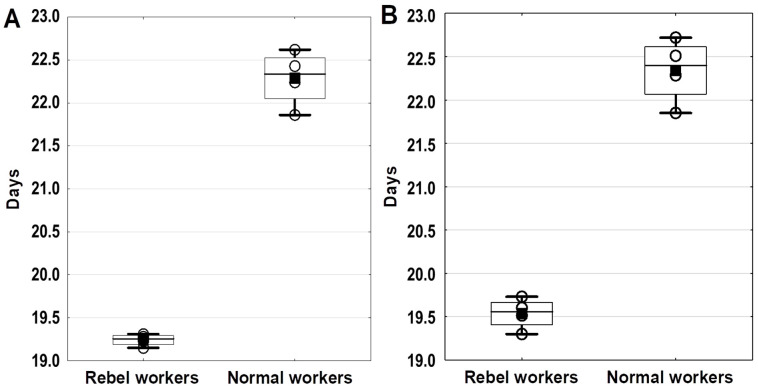
The total duration (days) of the preimaginal stages in the individual development of the first emerged workers (**A**) and the time necessary for 50% of the workers to emerge (**B**).

**Figure 2:**
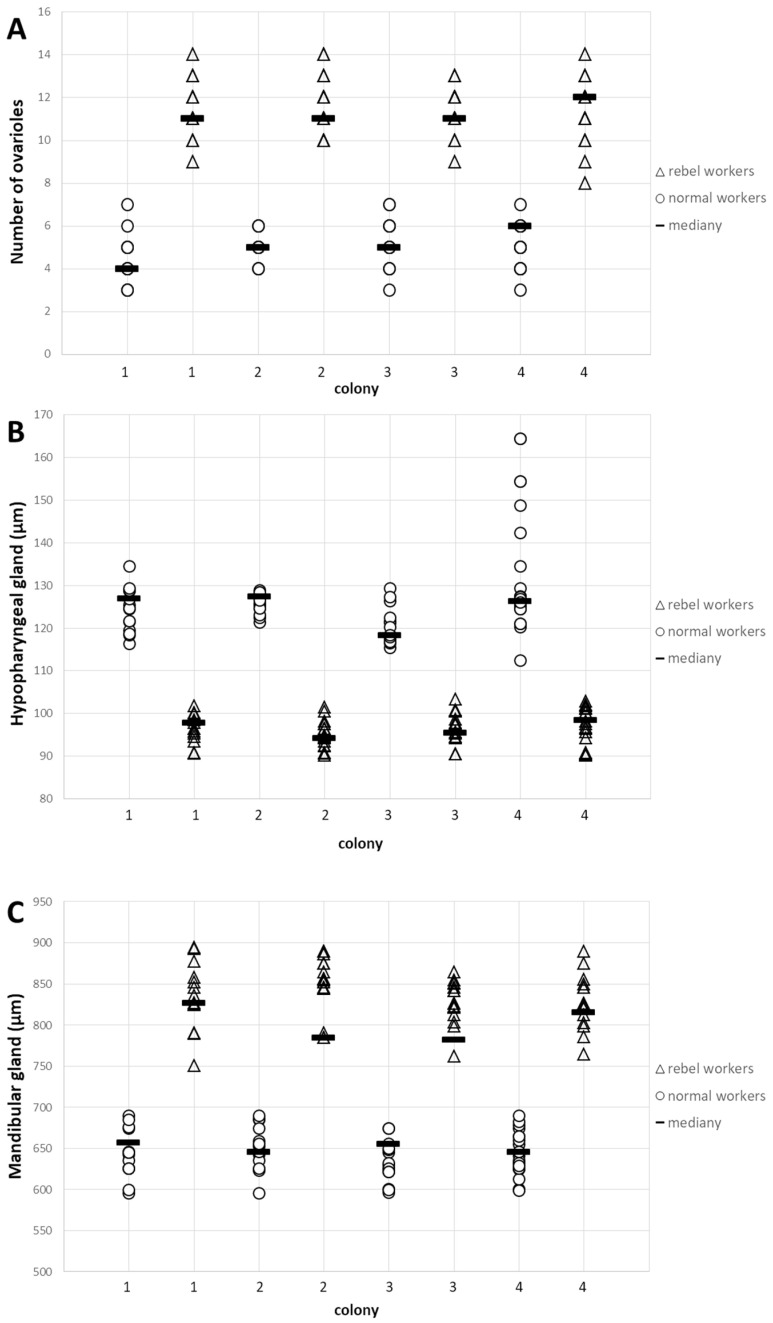
Anatomical characteristics of newly emerged honeybee rebel and normal workers. (**A**) number of ovarioles (colony: rebel workers *p* = 0.98, Kruskal–Wallis test; normal workers *p* = 0.08, Kruskal–Wallis test; rebel workers + normal workers *p* = 0.71, Kruskal–Wallis test), (**B**) size of hypo-pharyngeal glands (colony: rebel workers *p* = 0.36, Kruskal–Wallis test; normal workers *p* = 0.00, Kruskal–Wallis test; rebel workers + normal workers p = 0.12, Kruskal–Wallis test), (**C**) size of mandibular glands (colony: rebel workers *p* = 0.08, F3.66 = 2.36, ANOVA; normal workers *p* = 0.25, Kruskal–Wallis test; rebel workers + normal workers *p* = 0.21, Kruskal–Wallis test). No. of rebel workers = 70; No. of normal workers = 73.

The preimaginal stage durations of the rebel workers were shorter than those of nor-mal workers (Figure 1; four section combs). The rebel workers were characterized by larger numbers of ovarian tubules, reduced hypopharyngeal glands, and enlarged mandibular glands as compared to normal workers (*p* ≤ 0.01, Mann–Whitney test) (Figure 2).

The boxes indicate the data between the 25 and 75% quartiles, including the median (black line); the whiskers represent the minimum and maximum values; the black squares represent the mean; the wheels represent the number of section combs (*n* = 4); the differences between the rebel workers and normal workers are significant at *p* ≤ 0.05 (Mann–Whitney test). Total duration of the preimaginal stages of the first emerging workers were calculated by subtracting the date and time of the first workers’ emergence from the date and time of the queen’s caging on the comb sections; 50% worker emergence time point was calculated by subtracting the date and time of the workers’ exit from 50% of the brood cells from the date and time of the queen’s caging on the comb sections.

## 4. Discussion

We confirmed by analyses of numbers of ovarian tubules, hypopharyngeal glands, and mandibular glands that the emerged workers belonged to two sub-castes: rebel and normal workers (Figure 2), as in Woyciechowski and Kuszewska’s experiment [14]. The absence of a queen and her mandibular pheromones (QMPs) stimulates the emergence of rebels [10], which in their anatomical features are more queen-like than other workers and lay eggs. However, they do not avoid policing [22]. In initial studies, Kamakura [6] suggested that royalactin from royal jelly increased the body size and ovary development while shortening the development time in honeybees. The follow-up studies refuted the claims by Kamakura [23,24]. Royalactin has multiple functions: the monomeric MRJP-1 is necessary for basic growth and development and the oligomeric MRJP-1 binds with 10-hydroxy-2-decenoic acid to increase royal jelly viscosity [23,24]. Royalactin activates p70 S6 kinase, which is responsible for the increased activity of mitogen-activated protein kinase, and consequently associated with a shorter development time and an increased juvenile hormone titer—essential for ovary development [6,25]. Slater et al. [24] observed that it is the quantity, not the proportion of proteins and carbohydrates or water content, that has a significant influence on the final adult caste. Kucharski et al. [1] show that the caste switch is epigenetically regulated by silencing the expression of DNA methyltransferase Dnmt3, a key driver of epigenetic global reprogramming. In newly hatched worker larvae, this led to a royal jelly-like effect and increased the number of queens reared from worker larvae. Moreover, nutrition and differential gene expression are with the insulin/insulin-like genes, signaling cascades and the target of rapamycin (TOR) pathway [4,26]. This multi-pathway control is consistent with the concept of broad signal input [1,23]. Buttstedt et al. [23] also suggested that the food jelly of young worker larvae has very low sugar concentrations compared to the jelly of older worker larvae and to the royal jelly of all larval stages. The addition of sugar to worker jelly does not only affect the caste fate, but also increases the amount of worker jelly ingested by larvae to levels similar to that of royal jelly ingested by the queen-destined larvae. The queen determination is primarily driven by the amount of ingested food, thereby providing a higher amount of well-balanced nutrients for the developing queen larvae [23,27]. The question is whether rebel bees get royal jelly during their larval development for a longer time than workers in queen-right colonies or get food jelly with higher sugar concentrations and other well-balanced nutrients in comparison with normal workers. Another possibility is that the rebels get alternating food: royal jelly and food jelly. The next question is: which developmental stage, during larval development, during pupal development, or both, is shortened in rebels compared to normal workers? Obtaining an answer to these questions is crucial to clarify the shortened preimaginal development of rebel workers, experimentally confirmed for the first time here.

Vázquez and Farina [28] suggested that every phenotype results from the interaction between an individual’s genome and its context, which covers multiple parameters of both the environment, external to the organism, and the internal state of the organism, e.g., nutritional or hormonal state and the interaction among cells, tissues, and organs. In this sense, the context of early developmental stages is crucial for understanding the phenomenon of phenotypic plasticity. Wang et al. [29] suggest that one of the main factors influencing honey bee brood development is temperature. Honey bees regulate their nest temperatures with high precision. They spend considerable energy to maintain brood nest temperature in the range of 32–36 °C. In our apiary, we constantly monitored the temperature in the nest (compare to [30]), which was approx. 35 °C (both in C1 and C2 boxes) during the experiment. Therefore, we can consider this factor as not affecting the differences in the development period between the rebels and normal workers. Wang et al. [29] also list the following factors: the availability of royal jelly/food for the larvae, the presence of parasites and pathogens, and also pesticides. Since the boxes (C1 and C2) were connected to each other at the beginning and end of the experiment, it can be assumed that conditions in them were similar. Moreover, the colonies in our experiment were strong and of the same structure with a large number of nurse bees, which suggests the availability of food for the larvae. While we believe that none of these factors contributed to the variation in the length of the developmental period of female rebels and normal female workers, they should always be assessed and cannot be ruled out. Vázquez and Farina [28] report among these factors hormone-regulated processes that depend on the internal state of the brood. Additional research is needed to confirm the importance of hormones for the development of rebels. Therefore, we believe that our work, documenting the abbreviated period of development of the rebels, lays out directions for future research to identify the factors that influence this preimaginal development.

## Data Availability

The datasets generated during and/or analyzed during the current study are available from the corresponding author on reasonable request.

## Supplementary Materials

The following are available online at https://www.mdpi.com/article/10.3390/ani11113245/s1, Figure S1: The hypopharyngeal gland in a normal worker.
