# Supplementary material for: Reproductive Potential Accelerates Preimaginal Development of Rebel Workers in Apis mellifera
Source: Animals (Basel). 2021 Nov 13;11(11):3245. doi: 10.3390/ani11113245 (PMC8614343; doi:10.3390/ani11113245)
Supplement: Supplementary file 1 [file animals-11-03245-s001.zip › animals-1394219-supplementary.pdf]

Supplementary Material

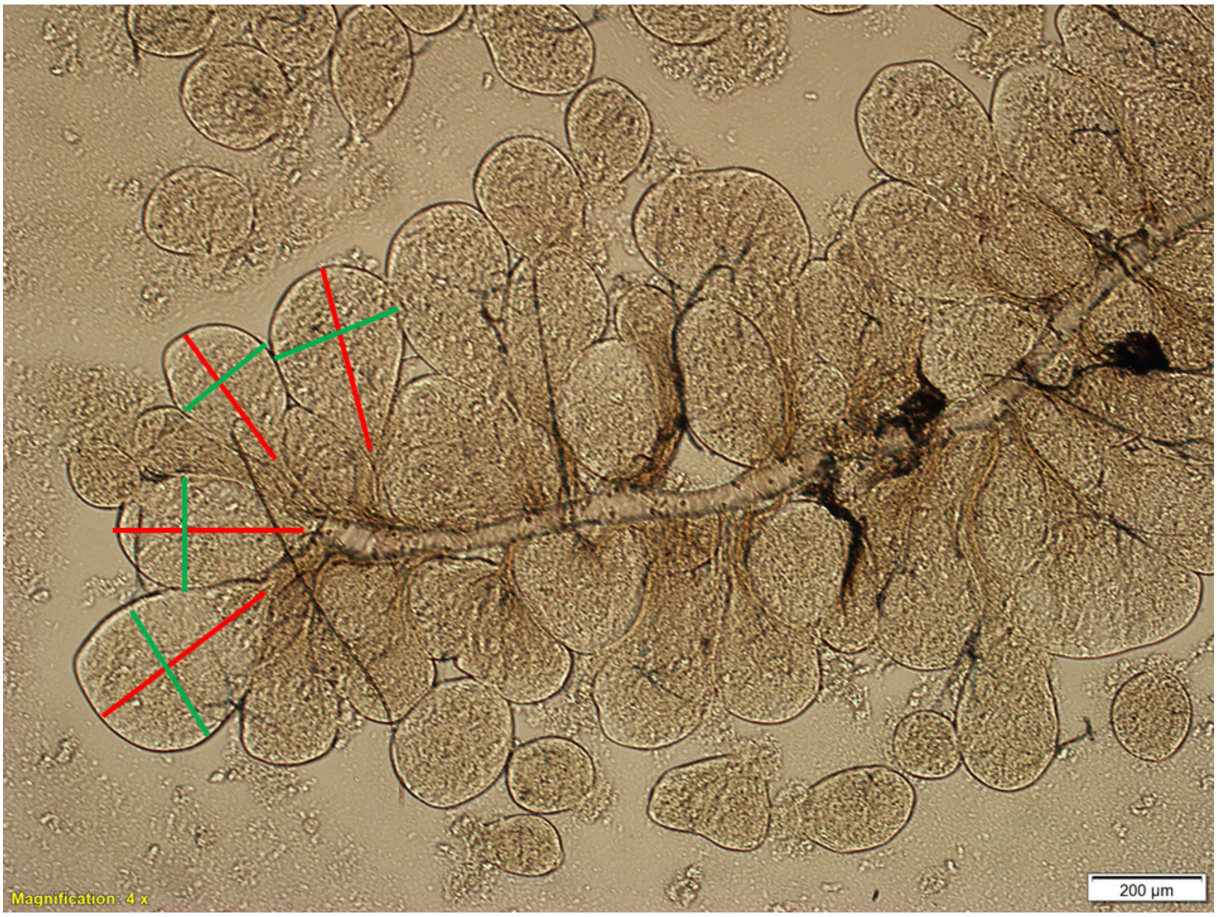

Figure S1. The hypopharyngeal gland in a normal worker. Red line - longest diameters, acini length; green line - shortest diameters, acini width.
